# Between (anti-)grammar and identity: a quantitative and qualitative study of hyperdialectisms in Brabantish

**DOI:** 10.1515/ling-2023-0148

**Published:** 2024-09-25

**Authors:** Kristel Doreleijers, Stefan Grondelaers

**Affiliations:** Department of Culture Studies, School of Humanities and Digital Sciences, 7899 Tilburg University, Tilburg, The Netherlands; Royal Netherlands Academy of Arts and Sciences, Meertens Institute, Amsterdam, The Netherlands

**Keywords:** hyperdialectism, dialect change, adnominal gender, acceptability judgments, quantitative vs. qualitative methods

## Abstract

Incomplete mastery of dialect grammar engenders ‘hyperdialectisms’ which may be unconscious errors, but which may also be the result of indexical resourcefulness, viz. the profiling of a regional identity. Fifty younger and older speakers from the Brabantish city of Eindhoven (Netherlands) were first administered an acceptability judgment task containing correct forms and three types of hyperdialectisms featuring gender and number constraints. Following the survey, the same respondents participated in a focus group discussion. Regression analysis on the scaled ratings revealed that all three types of hyperdialectisms were rejected, although it was especially the older respondents (almost all L1 dialect speakers) who were weary of the incorrect forms. Analysis of the focus group data demonstrated that older respondents are consciously aware of the rules of their dialect grammar, and hate it when these rules are violated. Younger respondents showed almost no meta-grammatical awareness, and admitted to ‘allowing’ the incorrect forms in some contexts because they ‘sound Brabantish’. Identity construction, in other words, is at the heart of hyperdialectal usage. Methodologically, this paper makes a plea for the confrontation of quantitative data – which provide the backbone of analysis – with qualitative data that offer access to motives for, and constraints on grammatical variation.

## 1 Introduction

Speakers who use a dialect they did not acquire as their first language regularly produce forms that deviate from the traditional dialect grammar. They inevitably make unintended, unconscious mistakes, but sometimes they deviate from the L1 grammar in a more conscious way for identity profiling purposes (e.g., Doreleijers 2023; Hoppenbrouwers 1990; Strandberg et al. 2022; Tsiplakou 2011). In both cases, a specific dialect feature is deployed in an unexpected way, leading to so-called *hyperdialectism* (e.g., Hinskens 2014; Lenz 2004; Trudgill 1988). The current paper deals with this concept of hyperdialectism in the context of the southern Dutch province of North Brabant. In this province, the local dialects (collectively labeled as ‘Brabantish’) still occupy a strong position, although they are subject to variation and change due to processes of dialect leveling and loss (Swanenberg and Van Hout 2013).

Traditionally, local dialects were used in domains of informal communication, whereas Standard Dutch was restricted to domains of formal communication. Nowadays, this diglossic situation has changed into a diaglossic situation, with the rise of intermediate variants across domains (Auer et al. 2005; Grondelaers and Van Hout 2011). Until the second half of the twentieth century, many people acquired a local Brabantish dialect as their first and home language, but due to increased mobility, the number of ‘native’ dialect speakers has declined sharply in the last decades and is expected to decline further (Hoppenbrouwers 1990; Swanenberg and Van Hout 2013; Versloot 2020). Convergence to Dutch has resulted in traditional dialects giving way to leveled varieties with a larger geographical reach, so-called regional dialects (Hoppenbrouwers 1990) or koines (Britain 2009).

However, these convergence processes are often accompanied by diverging tendencies amounting to an increase in dialectal variation (Doreleijers et al. 2020, 2021; Hinskens 2014). Speakers who attempt to achieve dialectal speech because “they do not know any better” (Trudgill 1988: 551), or because they want to portray a local identity (Doreleijers 2023), may overshoot their target and produce hyperdialectal forms. These speakers can be either L1 speakers deliberately aiming for a deviation from neighboring local dialects lacking the specific feature, L2 speakers or ‘semi-speakers’ (Dorian 1977) wanting to express their knowledge of the traditional dialect, but failing to do so because their analysis of the target variant is erroneous due to incomplete acquisition (Hinskens 2014: 114). In addition to this speaker-classification, the concept of hyperdialectism can also be divided into a structural and a statistical type (cf. Jamieson 2020). Structural hyperdialectism refers to the extension of a feature to contexts where it did not traditionally appear (e.g., Britain 2009: 135), whereas statistical hyperdialectism simply indicates (remarkably) high usage rates of a particular salient feature without extending it across contexts (Jamieson 2020: 18; Smith and Durham 2011: 218–219; Wolfram and Schilling-Estes 2006).

This paper exclusively focuses on structural hyperdialectisms and the ways they are evaluated by both older and younger speakers of the Brabantish dialect. Although hyperdialectisms *sound* dialectal, they actually stretch the grammatical boundaries of the dialect. For example, in current Brabantish dialect use, salient morphosyntactic features such as the diminutive suffix *-ske* (Swanenberg 2020) and the adnominal gender suffix *-e(n)* (Doreleijers et al. 2020) are overgeneralized, possibly because they are so divergent from Standard Dutch that they automatically conjure up a ‘Southern’ flavor. The use of the diminutive suffix *-ske* is traditionally only licensed when it follows velar sounds (e.g., *bankske* ‘little couch’) but it is also found, innovatively, following non-velar sounds (e.g., *clubske* ‘small club’). A similar phenomenon extends to the gender suffix for adnominal inflection, which is the main focus of the current study. The suffix *-e(n)* is traditionally restricted to singular masculine nouns (e.g., *enen hond* ‘a dog’ or *ene koning* ‘a king’) but in current hyperdialect it is also used with singular feminine or neuter nouns (e.g., *ene dame* ‘a lady’ or *ene koekske* ‘a cookie’), or even in definite articles preceding plurals (e.g., *d’n keuzes* ‘the choices’) (see also Section 2). One might wonder whether these hyperdialectisms have a real chance of becoming part of the dialect system, or whether they are just symptomatic of a languishing dialect. For older dialect speakers who speak the dialect as their first language, it is often assumed that hyperdialectisms are a source of annoyance, i.e., indicating dialect ‘decay’ or even dialect loss (Hoppenbrouwers 1990: 124), whereas younger speakers may perceive these hyperdialectisms as acceptable (Doreleijers et al. 2020; Jamieson 2020). However, robust evidence for this hypothesis that exceeds anecdotal observation and assumption is scant.

A commendable exception is Jamieson (2020), who investigated acceptability judgments by younger and older speakers of the Scottish Shetland dialect, a variety undergoing significant change. In Jamieson (2020), sentences containing hyperdialectisms were rated as more acceptable by younger speakers than by older speakers, and in this sense, they manifested “perceptual hyperdialectism”, i.e., hyperdialectisms observed within the confines of a metalinguistic task (Jamieson 2020: 21). Interestingly, Jamieson (2020: 21) argues that the younger speakers’ judgments do not seem to reflect their own grammar and usage. Instead, younger speakers substantiate their own intuitions with (unfounded) conjectures about more traditional (older or rural) grammars due to linguistic insecurity, i.e., the feeling that they are not able to perform the linguistic job at hand (Preston 2013: 324). The aim of the current paper is to investigate whether ‘new’ Brabantish dialect speakers are also showing evidence of perceptual hyperdialectism, and whether their presumed acceptance of deviant forms is the mere result of incomplete dialect acquisition or also of deliberate local identity-building.

The remainder of this paper is structured as follows. Section 2 describes variation in adnominal gender marking in Brabantish. The first part of this section sets out the three-gender system and the use of the suffix, whereas the second part focuses on hyperdialectal realizations of the suffix. Then, Sections 3 and 4 present a two-fold study that combines quantitative and qualitative methods in order to answer the research questions. Section 3 sketches the area of study, sets out the methodology used, i.e., an acceptability judgment task, and discusses the quantitative results to answer RQ1 and RQ2. Section 4 confronts the quantitative data with qualitative statements as obtained in focus group discussions featuring the same respondents as in the rating study; it describes the qualitative methodology and discusses the most revealing statements in answer to RQ3. Section 5, finally, provides some concluding remarks.

## 2 Adnominal gender marking and the notion of hyperdialectism

### 2.1 The main characteristics of the three-gender system in the Brabantish dialects

One of the main characteristics of the Brabantish dialects is the three-way gender distinction marked in the adnominal domain. This gender system has been eroded in the Dutch standard language from the seventeenth century onward due to a process of deflection, i.e., a decrease in linguistic cues which complicates the acquisition of the grammatical system (Audring 2009; Bloemhoff and Streekstra 2013; Tamminga 2013). Although Dutch nouns used to be either masculine, feminine, or neuter, the contrast between masculine and feminine has disappeared to a large extent, resulting in one class of common gender and a two-gender system that only distinguishes between common gender and neuter gender in the adnominal domain (i.e., on determiners and adjectives). However, in the southern Dutch varieties in the Netherlands, i.e., the dialects of North Brabant and Limburg, the system has persisted until now. The most salient feature within this gender system is the adnominal suffix *-e(n)* that precedes masculine singular nouns and is attached to determiners, such as definite and indefinite articles and adjectives (De Schutter 2013; Hoppenbrouwers 1990), e.g., *enen/den ouwen hond* ‘an/the old dog’ (m). This suffix enables speakers to overtly distinguish between masculine and feminine nouns within the category of common gender. Originally, the suffix is a remnant of Middle Dutch case-and-gender marking (Doreleijers and Swanenberg 2023b). Nowadays, the suffix only marks masculine gender in all cases. It functions as a variable with three variants *-e*, *-n*, or *-en* (see Table 1), whose selection depends on phonological constraints, to the extent that the so-called binding*-n* only precedes vowels or *h*, *b*, *d*, *t*. The different forms and contexts of usage are extensively described in traditional grammar descriptions, ranging from 1962 to 2013 (Doreleijers and Swanenberg 2023b).

**Table 1:** Overview of the different masculine suffix gender forms in (changing) Brabantish.

Form	Examples
(Non-dialectal) Standard Dutch forms	*de blauwe auto* ‘the blue car’(m) *een auto* ‘a car’ (m) *een bruine hond* ‘a brown dog’ (m) *de oude oma* ‘the old grandma’ (f) *een koekje* ‘a cookie’ (n) *de vrouwtjes* ‘the women’ (pl)
Compromise forms	*de* ***n*** *blauwe auto* ‘the blue car’ (m) *en* ***e*** *auto* ‘a car’ (m)
Traditional forms	*de* ***n*** *blauwe* ***n*** *auto* ‘the blue car’ (m) *en* ***en*** *bruine* ***n*** *hond* ‘a brown dog’ (m)
Hyperdialectisms	*de* ***n*** *ouw* ***en*** *oma* ‘the old grandma’ (f) *en* ***e*** *kuukske* ‘a cookie’ (n) *de* ***n*** *vrouwkes* ‘the women’ (pl)

However, processes of dialect loss and leveling have caused shifts within the traditional system. Already in the late twentieth century, researchers described the phenomenon of ‘hypercorrection’ which pertains to the production of overgeneralized forms, such as the masculine suffix preceding feminine nouns, e.g., *nen hillen ouwen oma* (f) instead of *’n hil aauw oma* ‘a very old grandmother’ (Hoppenbrouwers 1990: 123). It was assumed that “such ‘slippages’ leading to ‘superdialect’ only occurred in the usage of (young) informants who have not learned the gender distinction anymore” (Hoppenbrouwers 1990: 124, authors’ translation). Henceforth, hypercorrections have also been labeled ‘hyperdialectisms’ (e.g., Hinskens 2014; Lenz 2004; Trudgill 1988), as these forms do not reflect convergence to the standard language, but rather a deviation from the standard to emphasize the dialect. In any case, hyperforms are not the only non-traditional dialect forms of the gender suffix, as evidence of intermediate forms has also been found. These are called compromise forms (Hoppenbrouwers 1990; Swanenberg 2020), consisting of the masculine suffix *-e* without the binding-*n*, e.g., *en* ***e*** *hond* ‘a dog’. Therefore, these forms are to some extent indicative of a shift towards Dutch, although the dialect is still preserved in the partial application of the suffix. In previous research in the North Brabantish context, younger speakers (16–18 years old) were found to produce more hyperdialectal (ungrammatical) forms than traditional forms (Doreleijers et al. 2020): the masculine suffix was used not only with singular masculine nouns, but also with feminine and neuter singular nouns (e.g., *den ouwen oma* ‘the old grandmother’, *ene kuukske* ‘a cookie’), or even plurals (e.g., *den vrouwkes* ‘the women’).

The variation in the Brabantish gender marking system could be visualized as a continuum from dialect to standard language (cf. Lenz 2004), with the standard (non-dialect forms) at the ‘top’ and the traditional dialect at the ‘bottom’ of the continuum (cf. Auer 2011). While the compromise forms are intermediate forms which can be conceptualized as moving upwards to the standard, and moving away from the dialect, hyperdialectisms are intermediate forms which move *down* the continuum, and ‘push down’ the bottom, as it were, being amplifications (if not travesties) of the traditional dialect, to the extent that speakers producing them may *sound* dialectal but are actually overshooting the target. An overview of the different forms is presented in Table 1, in which boldface is used to index all non-standard forms.

### 2.2 The stratification of hyperdialectisms

An important consideration when it comes to studying hyperdialectisms is to what extent we can know for sure that we are dealing with hyperdialectisms (Doreleijers and Swanenberg 2023b). In other words: are hyperdialectisms merely a researcher’s construct to describe linguistic change that is not noticed by the speakers themselves, or are they a well-known linguistic phenomenon *inside* the community, engaged for identity work by some but abhorred by others? To improve our understanding of this issue, the concept of hyperdialectism can be modeled against the backdrop of Silverstein’s (2003) indexical orders, which more or less converge with Labov’s (1972) tripartite classification of indicators, markers, and stereotypes. The relevant question to ask in that respect is whether hyperdialectisms are the result of linguistic observation from the outside. Do they represent the linking of linguistic forms to specific groups who use them, as in the case of the researcher who ascribes hyperdialectal gender suffixes to young Brabantish (L2) speakers (in Silverstein’s taxonomy the latter would be a first-order indexicality). A second-order indexicality obtains when the hyperdialectisms are also noticed by the group members themselves, as a result of which they become available for conscious identity work. In the case of a third-order indexicality, a linguistic feature is considered so emblematic for a specific group that it is available for positive or negative meta-comments – for example the soft /g/ pronunciation for the southern Dutch (i.e., Brabantish and Limburgish) area (Cornips et al. 2017). Previous studies about Brabantish on social media have revealed that the masculine gender suffix has been enregistered as typically local, and can therefore be an asset in practices of place-making and stylization (Doreleijers 2023; Doreleijers and Swanenberg 2023a). (Digital) genres that aim at portraying the Brabantish identity, in particular in a humorous way, are privileged breeding grounds which frequently foster the use of hyperdialectisms such as the masculine gender suffix combined with non-masculine nouns. This suggests that speakers are at least in some cases aware of the dialect feature, for instance in metalinguistic reflections. Based on interview data, speakers have been found to be capable of producing forms that they consider grammatically ‘wrong’ to perform a Brabantish identity (Doreleijers 2023). According to the same reasoning, it could be equally possible, however, that hyperdialectisms go unnoticed in unstylized linguistic practices when identity construction is not, or to a lesser extent, at stake.

Another issue which complicates the use of the label ‘hyperdialectism’ is its overuse as an umbrella term for all sorts of deviations from the traditional dialect (Doreleijers and Swanenberg 2023b). Careful stratification of the concept can help avoiding such overinterpretations. First, when it comes to the hyperdialectal use of the gender suffix, it is possible to distinguish between phonological and morphological hyperdialectisms, or combinations of both (Doreleijers et al. 2020). In phonological hyperdialectisms, the use of the binding-*n* is overgeneralized, for example in masculine nouns that usually do not trigger it because of phonological constraints, e.g., *enen koning* ‘a king’, which begins with a consonant and hence does not require the binding-*n*. In morphological hyperdialectisms, the gender constraint is violated, e.g., *ene vrouw* ‘a woman’, and in phono-morpho hyperdialectisms, both constraints are violated, e.g., *enen vrouw*. Second, with this subdivision in mind, it is possible to further distinguish between other types of hyperdialectisms. These types are not only based on the way a specific form deviates from the traditional grammar, but also on the characteristics of the noun. For example, highly individuated nouns, i.e., nouns referring to animate persons and biological gender (to a lesser extent also feminine animals like cows), are most likely to be recognized as masculine or non-masculine by the speakers. In that respect, hyperdialectisms like *en* ***e*** *vrouw* ‘a woman’, which willfully ignore obvious gender indications, are more striking than hyperdialectal violations of nouns whose gender is less explicit (e.g., *en* ***e*** *tafel* ‘a table’ instead of *en tafel*). Another remarkable category consists of nouns with morphological endings indicating a diminutive, which are therefore grammatically neuter, e.g., *ene kuukske* (‘a cookie’) or *ene vrouwke* (‘a woman’), or nouns indicating plural number, e.g., *d’n mèskes* (‘the girls’). In the latter case, not only the gender constraint but also the number constraint is violated, as the gender suffix only precedes singular nouns in traditional grammar. In these categories, gender and number information is so obviously violated that said infringements are most likely to be consciously committed, plausibly for identity work.

In addition to hyperdialectisms concerning animates, diminutives, and plurals, there are other types of overgeneralizations of the gender suffix that are less straightforward in terms of hyperdialect. For example, for “lower” individuated nouns, i.e., nouns referring to objects or abstract entities, speakers are expected to be less sure of the lexical gender (Audring 2006). It is therefore much trickier to label these forms as hyperdialectisms, as speakers may not be able to establish the expected agreement between the suffix and the gender of the noun (Doreleijers and Swanenberg 2023b). Speakers who assume originally feminine nouns to be common (which are in Dutch generally referred to with masculine pronouns), such as *enen bank* (‘a couch’) or *ene fout* (‘a mistake’), cannot help producing seemingly hyperdialectal forms, because the underlying gender system is shifting. These unexpected forms are not necessarily violations of the gender constraint, but rather unconscious manifestations of this gender shift (although speakers of the traditional dialect who flawlessly master the three-way gender distinction may feel differently about it). Therefore, we will not consider these forms as hyperdialectisms in the remainder of this paper (and these are also not included in the experiment central to this paper).

### 2.3 Research questions

The previous overview leads to three research questions:

1. Are Brabantish sentences with hyperdialectal gender suffixes rated as less acceptable than Brabantish sentences with traditional gender suffixes?
2. Is there a difference in the acceptability judgments between younger and older speakers?
  Hypothesis: If hyperdialectisms are ungrammatical dialect variants which can be conscripted for identity service, they will plausibly manifest themselves in the idiolect of (younger) speakers who do not have the (incorrectly used) dialect as their L1 (or who haven’t completely acquired it as an L2), and plausibly also speakers who take pride in said dialect, even if they don’t speak it themselves.
3. If younger speakers should indeed be more tolerant for the ungrammatical variants, does this tolerance spring from limited proficiency, does it reflect the younger speakers’ own grammar and use (or what they think they know about the grammar and use of other [traditional] speakers [cf. Jamieson 2020]), or is there evidence for identity-related triggers?

With the aforementioned considerations (Sections 2.1 and 2.2) and research questions in mind, Section 3 will discuss the acceptability judgment task carried out in the current paper.

## 3 Acceptability judgment task

### 3.1 Method

#### 3.1.1 Background and context

The data for this study were collected between April and November 2022 in the city of Eindhoven and surrounding villages. With about 240,000 inhabitants, Eindhoven is the largest city in North Brabant and the fifth largest city in the Netherlands. Importantly, this region has been influenced by processes of urbanization, which is largely due to (labor) migration from other provinces and the influx of expats. Eindhoven is known for its high-tech industry with a global reach (hence its nickname *Brainport* city). These processes have put pressure on the local dialects, to pave the way for a more diverse linguistic reality, characterized by increasing dialect loss and leveling (Doreleijers et al. 2020; Wilting et al. 2014); the gender suffix is one of the dialect features particularly affected by attrition.

This urban context is an ideal research site to investigate hyperdialectisms, as both the decreasing number of native dialect speakers and language contact factors have been shown to be drivers of dialect change. Also, previous studies in the Eindhoven region have revealed that younger speakers are likely to produce hyperdialectisms at the expense of traditional forms (Doreleijers et al. 2020; Hoppenbrouwers 1990). The current study compares the acceptability rates of both correct forms and hyperdialectisms by both younger and older speakers in order to synchronically capture possible differences in the perception of the Brabantish dialect.

#### 3.1.2 Stimuli

The acceptability judgment task comprised 76 audio stimuli (including 12 fillers), featuring four types of dialectisms, which are presented in Table 2. In addition to correct forms (viz. regular gender markers in masculine singular contexts), we have included hyperdialectal violations of feminine animates, diminutives (neuter), and plurals, as these were shown to be the most straightforward in terms of the gender and number constraints. Thus, all nouns were easily recognizable as neuter due to the diminutive suffix, or as masculine or feminine due to biological gender. In total, 16 different nouns (see Table 2) were distributed among the four types of dialectisms. All singular nouns were presented in an indefinite context (preceded by the article *ene* or *enen* ‘a’). All plural nouns were presented twice in an indefinite context (preceded by the article-like adnominal *ginne* ‘no’) and twice in a definite context (preceded by the article *den* ‘the’).

**Table 2:** Four different conditions within the acceptability judgment task (*N* = 64).

Condition	Nouns included in the task
Condition 1. Masculine_expected form (*N* = 16)	*enen hond* ‘a dog’, *enen boer* ‘a farmer’, *ene vent* ‘a guy’, *enen Braobander* ‘a *Brabander* (male person from Brabant)’
Condition 2. Feminine_hyperdialectism (*N* = 16)	*enen dame* ‘a lady’, *enen dochter* ‘a daughter’, *ene koe* ‘a cow’, *enen buurvrouw* ‘a (feminine) neighbor’
Condition 3. Neuter_hyperdialectism (*N* = 16)	*ene kuukske* (‘a cookie’), *ene bèkske* (‘a little bowl’), *ene clubke* (‘a little club’), *ene vrouwke* (‘a woman’)
Condition 4. Plural_hyperdialectism (*N* = 16)	*ginne/den bluumkes* (‘the little flowers’), *ginne/den fisjes* (‘the parties’), *ginne/den mèskes* (‘the girls’), *ginne/den kassières* (‘the cashiers’)

All stimuli were produced by a 38-year-old native speaker of an East-Brabantish dialect. This speaker comes from the Eindhoven region and has worked for more than ten years as a teacher at a primary school in the small town of Sint-Oedenrode. Although both our respondents and our experimental speaker come from the Eindhoven area, we used a variety of Brabantish that was somewhat more general. Local variants in pronunciation or (rarely used) vocabulary were consciously avoided to maximize the likelihood that lower acceptability rates would not be caused by speakers being unfamiliar with the dialect used in the task. In order to ensure that the Brabantish in the test sentences would be ‘natural Brabantish’ (instead of Dutch with a Brabantish accent and gender suffix), a number of other salient Brabantish dialect features, i.e., shibboleths, were distributed over all stimuli. For example, the possessive pronoun *ons* ‘our’ (*ons Marie*) to indicate a relative is considered typically Brabantish, just like the second person verb form in inversed sentence structures, *wilde* instead of *wil je* ‘would you’ and *kunde* instead of *kun je* ‘can you’. Also, second person pronouns *gij* and *ge* ‘you’ and the possessive pronoun *oew(e)* ‘your’, are well-known Brabantish dialect features. Moreover, non-standard pronunciations, such as *hèt* instead of *heeft* ‘has’, *ao* instead of *aa* (e.g., *straot* ‘street’), *dè* instead of *dat* ‘that’, *vanmèrge* instead of *vanmorgen* ‘this morning’, and *meej* instead of *met* ‘with’, give the language a clearly recognizable Brabantish flavor. In this way, we ensured that all stimuli radiated a genuine dialectal Brabantishness that was not, however, limited to a very specific location.

Examples of the test items are presented in (1–4); while (1) contains an expected form, i.e., a masculine singular noun with a masculine gender suffix (condition 1), the examples in (2–4) are hyperdialectal (conditions 2–4). In Examples (2) and (3) the gender constraint is violated (a feminine (2) or neuter (3) noun with a masculine gender suffix), and in Example (4ab) the number constraint is violated (a masculine gender suffix for singular nouns with a plural noun).

(1)

**Table:** 

*Vanmèrge*	*heb*	*ik*	*en-**en** hond*	*uitgelaote.*
This morning	have	I	a-m.sg dog.m.sg	walked
‘This morning I walked a dog.’

(2)

**Table:** 

*Ons Marie*	*hèt*	*gister*	*en-**en** dochter*	*gekrege.*
Our Marie	has	yesterday	a-m.sg daughter.f.sg	got
‘(Our) Marie gave birth to a daughter yesterday.’

(3)

**Table:** 

*Wilde*	*gij*	*graog*	*en-**e** kuukske*	*bij*	*oewe koffie?*
Would	you	like	a-m.sg cookie.n.sg	with	your coffee
‘Would you like a cookie with your coffee?’

(4)

a.

**Table:** 

*Ge*	*ziet*	*in*	*dè durp*	*ginn-**e** mèskes*	*op straot.*
You	see	in	that village	no-m.sg girls.n.pl	on the street
‘You don’t see girls on the street in that village.’

b.

**Table:** 

*Meej*	*carnaval*	*kunde*	*de-**n** mèskes*	*versiere.*
With	carnival	you can	the-m.sg girls.n.pl	chase
‘With carnival you can chase the girls.’

#### 3.1.3 Respondents

In total, 50 respondents in two age groups (25 aged 16–18; mean: 16.6, and 25 aged 50+, mean: 72.5) took part in this study. Younger participants were recruited through a school for secondary education (senior general and pre-university level) in the city center of Eindhoven. All young respondents were selected based on teacher recommendations pertaining to individual respondents’ dialectal way of speaking and their membership in a peer group of close friends that could take the experiment together and would be available afterwards for a focus group discussion. In addition, participation was restricted to respondents who had grown up in the Eindhoven region, and who still live there. We did not, however, restrict our younger sample to respondents who had grown up exclusively with a Brabantish dialect: they were required to have a clearly recognizable Brabantish way of speaking, in terms of accent, lexicon, or grammar. In short, we tried to compose a younger respondent panel that was representative for the situation of dialect loss and leveling in Eindhoven. For some of the younger respondents this criterion materialized in regionally flavored Dutch, whereas others reported that they spoke an intermediate variety between dialect and Dutch. Eventually, one younger respondent was removed from the sample because he reported an L1 other than Dutch or a Dutch dialect (Bulgarian), and one because gender information was not provided. Of the 23 young respondents eventually included, 14 were male and 9 were female, 16 reported to speak Dutch as their L1, 6 reported to speak an intermediate variety, and 1 was a self-reported dialect speaker.

Older participants were recruited through the network of the provincial heritage organization *Erfgoed Brabant*. Five L1 dialect speakers were approached by e-mail by the researchers to participate in the study. Each of these contacted four dialect-speaking relatives or friends to join them for the experimental session and the subsequent focus group discussion. All older participants came from five different villages near Eindhoven, i.e., Dommelen, Valkenswaard, Nuenen, Lieshout, and Best. Two older respondents were excluded from the analysis, one because he reported an L1 other than Dutch or a Dutch dialect (English), and one because she skipped an entire question on dialect use outside her family. Of the 23 older respondents eventually included, 15 were male and 8 were female, two reported to speak Dutch as their L1, while 21 reported to be native dialect speakers. This apparent imbalance in dialect knowledge and acquisition between the two groups (younger vs. older speakers) is representative of the current situation in the Eindhoven region, where mainly the older speakers still grew up with dialect as their first language, while youngsters acquire the dialect as a second language later in life.

Two researchers (both familiar with the Brabantish dialect, one as a native speaker and one as an L2 speaker, i.e., the first author of this paper) conducted the experiment in ten collective sessions which each featured five younger or five older speakers.1Jos Swanenberg from the Meertens Institute (KNAW), the Netherlands, joined the first author of this paper in the research sessions. He is also a native speaker of the dialect. The sessions with the younger participants took place in a school classroom, whereas the sessions with the older speakers took place at a location chosen by the participants themselves, viz. twice at the homes of participants, twice at a local history center, and once at a dialect center. All participants were informed about the study by means of a letter one week prior to the study. This information letter contained information about the general topic of the study, i.e., Brabantish, but it did not, of course, disclose the actual research goals – hyperdialectisms and the gender suffix. Informed consent was obtained at the beginning of each session: all respondents assented to being recorded on audio and video for the final part of the study (focus group discussions). Participants were told that they could terminate the study at any given point.

#### 3.1.4 Procedure

The study consisted of three parts which lasted for about 60 min in total. In the first part, respondents provided demographic data on their age, gender, place of birth, place of residence, and linguistic profile (including their L1, self-reported dialect proficiency, dialect use in and outside their family, and attitudes, e.g., “Proud of Brabantish”). In the second part of the study, which took approximately 20 min, participants carried out an acceptability judgment task in a within-subjects design. All participants were given a predesigned data form, and they were asked to rate the 76 sentences, played as audio stimuli, on Likert scales ranging from 1 (‘totally unacceptable’) to 5 (‘totally acceptable’). Participants were instructed to ignore small phonological or lexical differences which differed from their own way of speaking. Instead, they were asked to judge whether they considered the sentence to be grammatically acceptable or not. To make sure participants understood this assignment, they were given four trial stimuli to acquaint themselves with the experimental set-up and the stimuli, and they were given the opportunity to ask questions. In addition, we asked them why they had assigned low or high ratings to the trial stimuli, without revealing that we were in fact looking for their ratings of the gender suffix. During the trial stimuli as well as in the actual task, each sentence was presented only once to engender fast, unpremeditated responses.

In the third part of the study, all respondents participated in a focus group discussion. The method of this qualitative part of the study will be explained in Section 4 below. Participants did not receive any financial reward for their participation in the study. However, the first author provided guest lectures and feedback on students’ research presentations in the school classes involved in this study. Older participants were given a popular scientific book about dialects (co-authored by the researchers) as a reward.

In Section 3.2 the quantitative results of the judgment task will be presented.

### 3.2 Quantitative results

In view of the ordinal nature of our five-point Likert scale, and the fact that we wanted to include Respondent and Noun as random factors, we fitted a cumulative link mixed model on our ratings (package ordinal in R). The following predictors were considered for inclusion in the model. In addition to the fixed effect Dialectism (Correct [Cor] vs. Plural [Plu] vs. Singular Neuter [SingN] vs. Singular Feminine [SingF]), we entered a large number of covariates in the modeling, including the demographic factors respondent Gender (M vs. F), Age Group (Young vs. Old), and L1 (Dialect vs. Intermediate vs. Standard Dutch). In addition, we included several usage variables such as Mean Contact with Brabantish (1 “never” to 5 “on a daily basis”), Frequency of intra-familiar use of Brabantish (1 “never” to 5 “on a daily basis”), and Frequency of use of Brabantish in other contexts than one’s family (1 “never” to 5 “on a daily basis”). A final set of covariates included the perceptual question how Brabantish-sounding respondents found their own speech (Not at all vs. Somewhat vs. Very much) and the Attitudinal question how “Proud of Brabantish” respondents considered themselves (see under Procedure, 1 “not at all” to 5 “very much so”). As random factors, we included the ID of the Respondent, and Noun type. All main effects and two- as well as three-way interactions were considered. Please see the Appendix for a table with the raw counts for all stimuli, describing the number of participants opting for the different points on the Likert scale per stimulus.

The most parsimonious model (AIC 7513.77; Marginal *R*^2^ = 22.7; Conditional *R*^2^ = 50.4) contained a significant main effect of Dialectism, a near-significant main effect of Age, as well as a significant interaction between Dialectism and Age. Compared to the reference level Dialectism = Correct, the levels Plural (Odds Ratio 1/0.039 = 25.6; *p* < 0.0001), SingN (Odds Ratio 1/0.037 = 27.02; *p* < 0.0001) and SingF (Odds Ratio 1/0.053 = 18.9; 0 < 0.0001) are all deemed significantly less correct. Odds ratios are effect size estimates which should be interpreted as follows. Negative odds ratios indicate that perceived grammaticality decreases on account of a main effect or interaction; the negative odds of 0.037 for the singular neuter hyperdialectism, for example, reveal that the odds for perceived unacceptability increase 1/0.037 = 27.02 times when a stimulus contains a singular neuter hyperdialectism, compared to when it contains a correct form. The unacceptability odds for the plural hyperdialectism (25.6) and the singular feminine hyperdialectism (18.9) equally index strong rejection.

**Figure 1:**
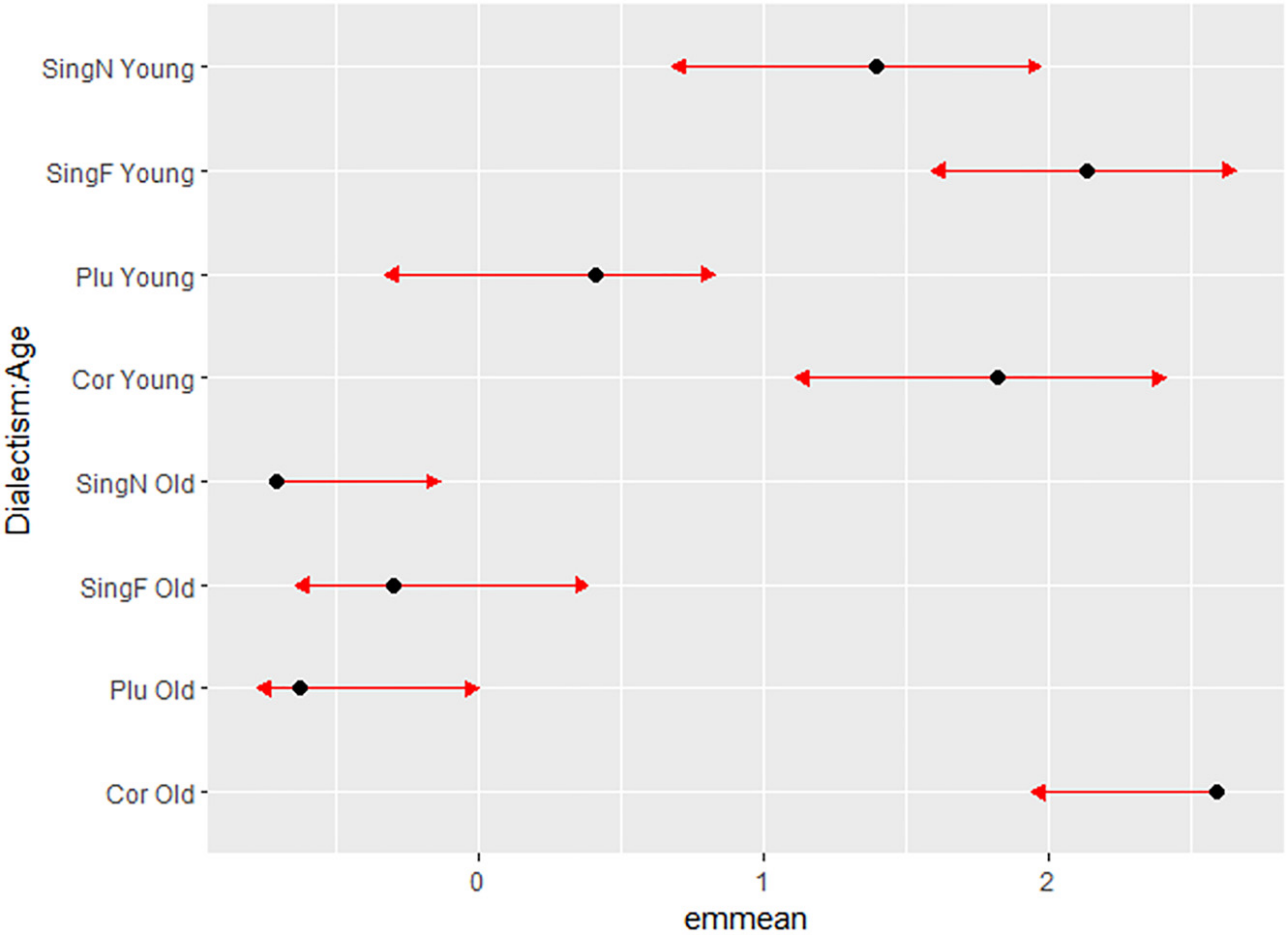
Estimated marginal means and comparison arrows for interaction Dialectism × Age.

The significant interaction Dialectism × Age (*p* < 0.0001), however, reveals that these effects are restricted for the most part to *older* respondents. For convenient interpretation of the interaction, we computed pairwise comparisons based on estimated marginal means (package emmeans in R). These are plotted in Figure 1.

In the plot in Figure 1, the comparison arrows framing the estimated means can be used to determine whether levels are significantly different from one another: if an arrow from one mean overlaps an arrow from another mean, the difference is not significant, based on the adjust setting (“Tukey”) and the alpha value (0.05).2https://cran.r-project.org/web/packages/emmeans/vignettes/comparisons.html. https://cran.r-project.org/web/packages/emmeans/vignettes/comparisons.html. As can be inferred from the (non-)overlapping arrows in Figure 1, the difference between older and younger evaluations does not pertain to all four Dialectism levels: older and younger respondents only differ in their evaluation of SingF (−2.44; *p* < 0.0001) and SingN (−2.1; *p* < 0.0001); their judgments of Cor (0.77; *p* = 0.58) and Plu (−1.04; *p* = 0.17) overlap and are not significantly different.

If we interpret the interaction from the perspective of age groups, it is clear that older respondents are more categorical in their rejection of all hyperdialectisms. This is indexed, to begin with, by the smaller arrow widths, which signal more evaluative certainty, but also by the large distance between evaluations of the Correct form on the one hand and the hyperdialectisms on the other (all *p* < 0.0001 in the paired comparisons). Older respondents' evaluations of the three hyperdialectisms, however, are not significantly different (all ns). In the younger group, confidence intervals are much wider, and overlap for Correct, SingN, and SingF, so they signal no significant differences in the evaluations (all ns). The only significant differences in the younger evaluations pertain to the lower perceived grammaticality of plural hyperdialectisms with respect to correct forms (−1.41; *p* < 0.0001) and with respect to singular feminine hyperdialectisms (−0.98; *p* = 0.022).

**Figure 2:**
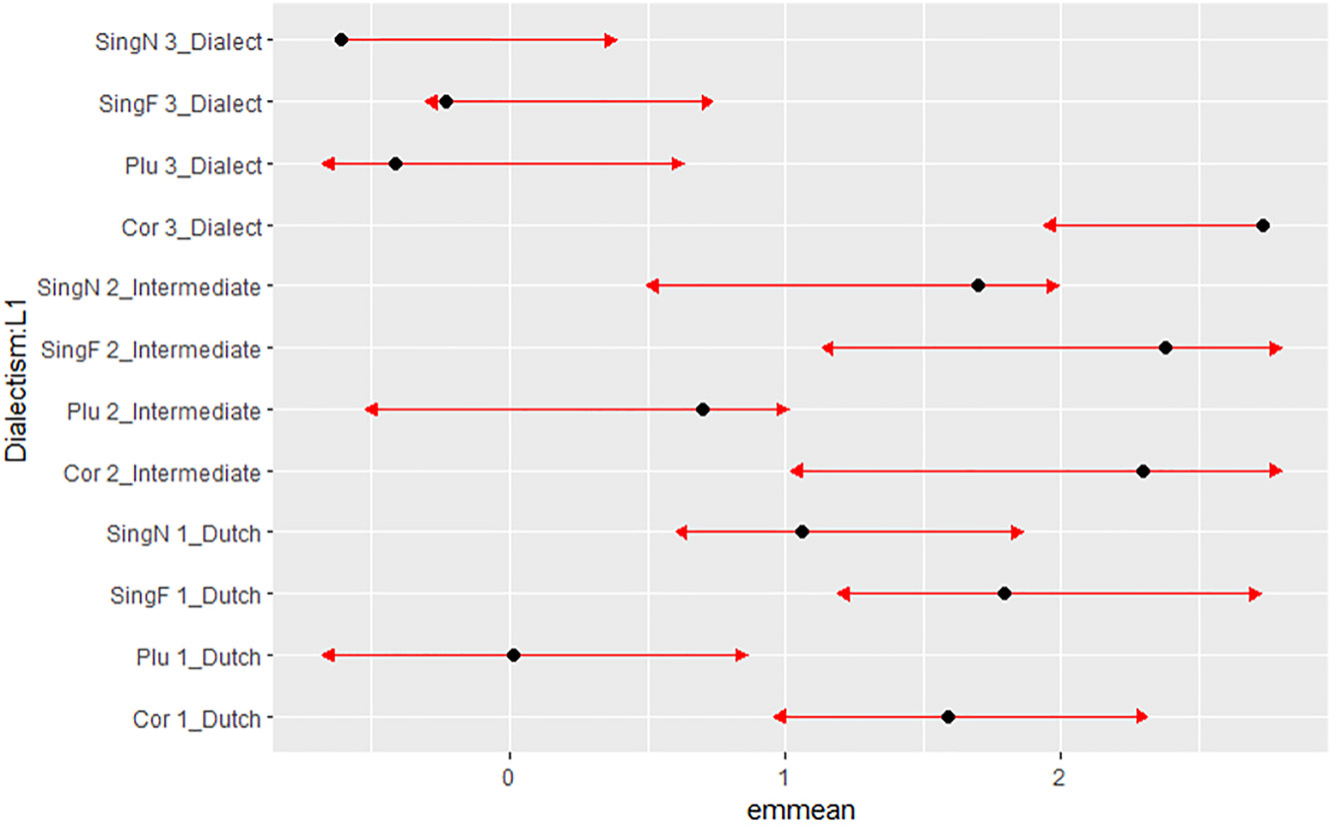
Estimated marginal means and comparison arrows for interaction Dialectism × L1.

It should be noticed that the difference between older and younger evaluations partly masks an L1 effect: recall that 21 out of the 23 older respondents are L1 dialect speakers, while only one younger respondent is a native dialect speaker. Although variation inflation factors (Age = 1.29; L1 = 1.17) do not suggest multicollinearity, the two factors apparently are so correlated that L1 was not selected in the best model.3An anonymous reviewer suggested creating a new variable that combines Age and L1. However, we did not do this because we do not feel that this does credit to the conceptual approach underlying our experiment: comparing acceptability judgments from younger and older speakers from the same region to gain insight in age-related (apparent time) perceptions of hyperdialectisms. For us, Age and L1 are of course related, but they do not have a similar status; we are primarily interested in how older and younger speakers from the same region make sense (of) their dialect; in the case that they are not L1 proficient in the dialect, our second question is how they use the dialect (for instance to make sense of their regional identity). A second model that we fitted considered Dialectism and L1 as main effects and in interaction. That second model was weaker than the best model (7,535.41 < 7,513.7); it contained a main effect of the plural dialectism (*p* < 0.0001), a near-significant effect of the singular neuter dialectism (*p* = 0.0859), but a significant interaction (*p* < 0.0001) between Dialectism and L1 = Dialect, but not between Dialectism and L2 = Intermediate or L2 = Dutch. For convenient interpretation, we once more resort to a plot of the estimated marginal means in Figure 2.

As can be inferred from the comparison arrows in Figure 2, categorical rejection of all three types of hyperdialectisms is restricted to L1 dialect speakers: while the plural hyperdialectisms are globally rejected, speakers of Dutch or an Intermediate variety do not consider the singular neuter or feminine forms as less acceptable than the correct forms.

### 3.3 Discussion of the quantitative results

The just reported data permit us to answer RQ1 and RQ2. Hyperdialectisms are rated significantly less acceptable than traditional dialect forms (RQ1). In addition, the results reveal a clear pattern of behavior based on age (cf. Jamieson 2020), as the main effect of Dialectism is for the most part restricted to the older participants (RQ2). This age effect, however, is also an L1 effect, to the extent that the majority of older speakers are L1 dialect speakers, and only these speakers categorically reject all hyperdialectisms.

Do these differences spring from the fact that older (dialect) speakers master the traditional rules of dialect syntax, whereas the youngsters do not fully master them and may therefore consider all forms (whether correct or hyperdialectal) acceptable? The latter does not appear to be the case as both younger and older respondents rated plural hyperdialectisms as less acceptable. These forms do not (only) violate the gender constraint (i.e., the suffix should only be combined with *masculine* nouns), but rather the number constraint (i.e., the suffix should only be combined with *singular* masculine nouns). In previous research on Brabantish on social media, the gender suffix was found multiple times with plural nouns (Doreleijers 2023). Nonetheless, the results of the acceptability judgment task indicate that these forms are likely to be the ‘next stage’ in dialect change that only comes after a shift from masculine gender to *all* types of gender, i.e., from (1) masculine singular nouns to (2) singular nouns (regardless of their gender) to (3) all nouns (regardless of their gender and number). This presumed shift is supported by the quantitative results, as there is no significant difference between the younger respondents’ ratings of the correct forms and their ratings of the feminine and neuter hyperdialectisms. This finding indicates that the younger respondents are moving away from the original grammatical function of the gender suffix, a tendency which is also supported by the qualitative evidence to be discussed in Section 4. We also turn to qualitative evidence in order to answer RQ3, which pertains to the how and especially the why of hyperdialectisms: does the younger respondents’ tolerance for these deviations spring from a lack of proficiency (as the quantitative data suggest), does it reveal a reliance on other grammars than their own, possibly deficient ones, and/or are the incorrect features conscripted for identity work?

## 4 Qualitative analysis of the focus group data

### 4.1 Method

**Figure 3:**
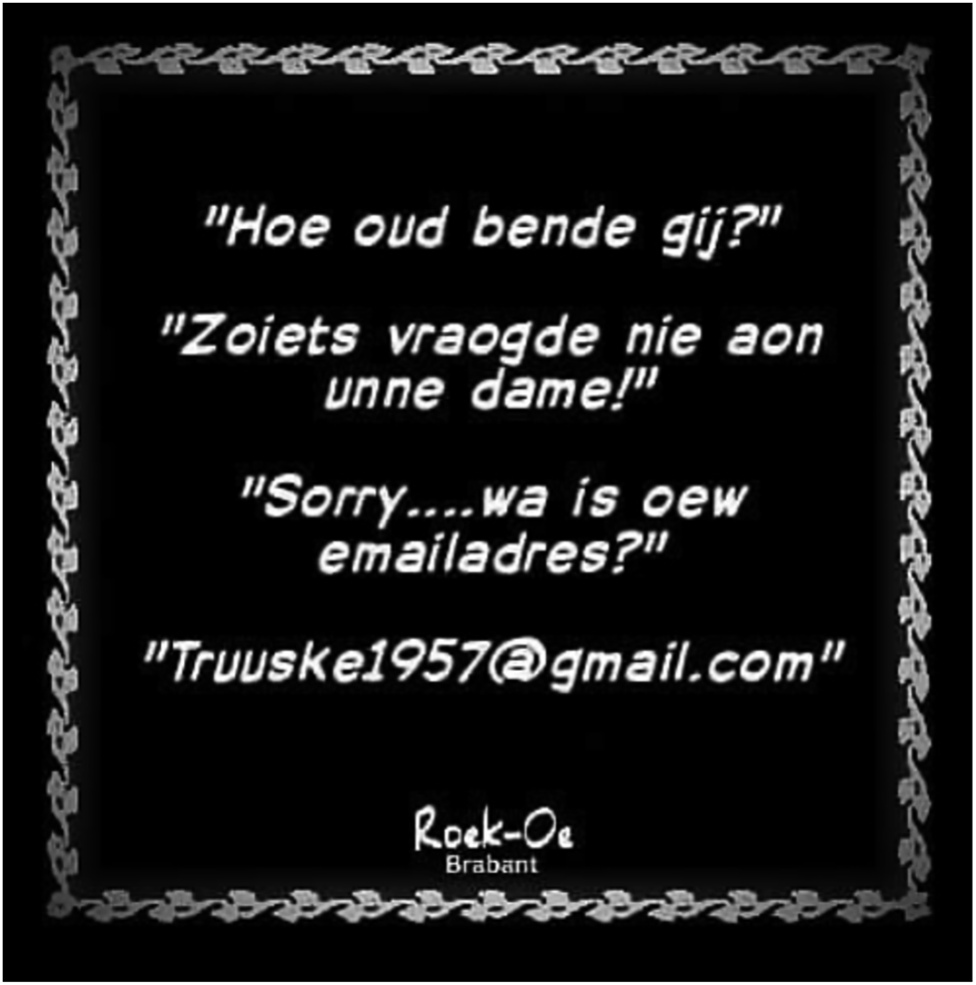
Prompt with hyperdialectal *unne dame* ‘a lady’.

In the third part of the study, participants took part in a focus group interview which we organized to obtain some qualitative ‘flesh’ on the quantitative spine of the survey results. In this interview, they were confronted with three different prompts in a row, viz. pictures containing hyperdialectisms pertaining to the gender suffix, to which they had to respond individually and all together by means of a group discussion. An example is displayed in Figure 3. This picture was taken from the Instagram page of *RoekOe Brabant* (cf. Doreleijers 2023) which aims to depict Brabantish culture and identity, and consists of a fictitious dialogue between Truuske (female) and an undefined male person, asking her about her age (*Hoe oud bende gij?* ‘How old are you?’). Truuske replies in an offended manner: *Zoiets vraogde nie aon unne dame!* ‘You don’t ask a lady something like that!’ This is followed by the male person asking for her e-mail address, and in her answer (the e-mail address) Truuske gives away her age after all. The text contains multiple dialect features, including a hyperdialectism in which an animate feminine noun *dame* ‘lady.f.sg’ is preceded by the masculine gender suffix on the indefinite article *ene*, here written as *unne* ‘a-m.sg’ (cf. condition 2 in Table 2).

In each focus group discussion (ten in total: five with the younger participants and five with the older participants), the researchers started the conversation by asking the same question: ‘Is this picture Brabantish to you?’ Then, the participants could take the lead to start talking, and because the groups consisted of peers, a natural flow in the conversations quickly developed. Meanwhile, the researchers kept an eye on ensuring that each participant had sufficient opportunity to speak, and, if necessary, they asked a question directed at a specific individual to include them in the discussion. Also, the researchers joined the discussion by asking specific questions to guide the conversation or to clarify at some point. To keep the main topics across the different focus groups as consistent as possible, i.e., to ensure they were discussed in a similar fashion and in the same order, the researchers used a topic guide (cf. Matthews and Ross 2010: 246).

For the current study, the first and last part of the focus group discussions are of particular interest. In the first part, participants had to discuss the three prompts in terms of ‘Brabantishness’, the linguistic features that contribute to an impression of Brabantishness, and authenticity. In the final part, the researchers reintroduced the prompts to specifically ask the participants about the usage of the gender suffix in the pictures (for the first time in the study). In general, this part was guided by the question whether participants know when to use the Brabantish gender suffix instead of the Standard Dutch forms. The lingua franca of all discussions was Dutch, but occasional interference of the Brabantish dialect was allowed. Each focus group discussion lasted 31 min on average, and was transcribed afterwards. The transcribed data were analyzed manually by means of a thematic analysis (Braun and Clarke 2006). In total, the researchers identified 20 different themes (see Doreleijers and Swanenberg [forthcoming] for an overview and in-depth discussion). For the current paper, the juxtaposition of the younger and older participants lies at the heart of our qualitative analysis.

In the next subsection, key statements (in light of RQ3) from the focus groups will be presented and discussed simultaneously.

### 4.2 Qualitative statements

While the quantitative data show clear patterns in the ratings of correct and hyperdialectal forms (RQ1), and in the impact of respondent age on the ratings (RQ2), they still leave RQ3 unanswered, especially when it comes to the following three topics: (1) speakers’ explicit awareness of the dialect grammar, (2) the dialect knowledge that is reflected in the ratings, and (3) the identity work that is at stake. To fill this gap, qualitative statements were collected from specific parts of the focus group discussions, namely the part that explicitly questions participants about the acceptability of using a masculine gender suffix with an (animate) feminine noun (Figure 3), and about the way this hyperdialectal use contributes to authentic ‘Brabantishness’. Also, the final part of the discussion, where participants were asked about their knowledge of the gender marking rule, provides some relevant information. In the remainder of this section, the statements are presented in three subsections.

#### 4.2.1 Explicit awareness of the dialect grammar

The big contrast between the younger and older participants resulting from the acceptability judgment task is also reflected in their respective awareness of the dialect grammar. Relying on their ‘linguistic feeling’ and traditions, *all* older participants consider hyperdialectisms to be violations of the traditional grammar, with some of the older participants even being able to explicitly state the lexical gender marking rule (the gender constraint, or even the phonological constraint), which is for example shown in the following statements. Note that participants O1B, O1D, and O3B are all L1 speakers.4Please note that the pseudonyms consist of the age group of the participants (O = Older, Y = Younger), the number of the focus group (1–5), and the individual participant within each group (A–E). R indicates the Researcher (i.e., Interviewer). Participant O5B, however, is an L2 dialect speaker who reported Dutch as his L1. Nonetheless, this speaker has acquired a high dialect proficiency at a later age.

**Table:** 

O1B:	[…] *’ne mens*, da zegde wél. Mar nie […] *’n* ***e*** *daome*.
	‘*’ne mens* “a man” you *do* say. But not *’ne daome*.’
O1D:	Mannelijk of vrouwelijk.
	‘Masculine or feminine.’
O3B:	’t Is ’n kwestie van is ’t is ’t eh zelfstandig naamwoord, is det ’n mannelijk of vrouwelijk?
	‘It’s whether the noun is masculine or feminine?’
O5B:	*D’n* is geslachtsafhankelijk en dè kan alleen mar bij mannelijke woorden, en dan ok nog alleen as, eh, dit begint as, eh, dit begint wel met ’n *b* […] ’t is wel *d’n boer*, dè klopt wel, want boer is mannelijk …
	‘*D’n* depends on lexical gender, and it can only combine with masculine words, and only if it [the noun] begins with eh, this [noun] begins with a /b/, it’s *d’n boer* ‘the farmer’, that’s correct, because *boer* is masculine …’

The younger participants completely lack this traditional grammar knowledge. Instead, they propose different kinds of norms that are not related to lexical gender at all. For example, participant Y2A and Y2D (both L2 speakers of the dialect) relate the use of the suffix to a situational context, and are thereby adhering to an innovative stylistic norm instead of the traditional grammatical norm.

**Table:** 

Y2A:	Ik gebruik het denk ik vaker als ik een beetje dronken ben. Als we dan weet ik veel ’s avonds met een paar vrienden in het café staan.
	‘I think I use it more often when I’m a little drunk. When we’re in the bar at night with a couple of friends.’
Y2D:	Ik pas het denk ik best wel aan aan de setting waarin ik ben en met welke mensen.
	‘I think I pretty much adapt it to the setting in which I am and with which people.’

There are also younger participants, all L2 dialect speakers, who do not have any sense of a norm, although they *do* think that the suffix *can* be used incorrectly. These participants are rather stuck with the adage of ‘just do it’ (i.e., just use it). Participant Y3B, the only younger speaker who reported the dialect as her L1, indicates to use the suffix automatically without being able to explain the underlying grammatical rule.

**Table:** 

Y2C:	Ik denk da er nie veel regels achter zitten.
	‘I think there are not many rules behind it.’
Y2A:	Gewoon doen.
	‘Just do it.’
Y2C:	Je komt er vanzelf wel achter als het niet klopt.
	‘You will find out if it’s not correct.’
Y3B:	Ik weet niet of ik ’t zo kan uitleggen maar meestal gebeurt ’t vanzelf ofzo.
	‘I don’t know if I can explain it this way but usually it happens automatically or something like that.’

Interestingly, participant Y2C (who reported an intermediate variety between the dialect and Dutch as his L1) turns out to be very certain about the hyperdialectal form *unne dame* being the correct dialect form, while considering the use of the gender suffix a bit redundant in some of the audio recordings.

**Table:** 

Y2C:	Da was ook met bij een van die spraakmemo’s […] dan zeggen ze extra tussendoor nog een keer *unne* wa dan ’n beetje overbodig is als je da nu zou zeggen.
	‘This was also the case with the audio recordings […] then they say one more time *unne* in between, which is a bit redundant if you would say it now [in the conversation].’
R:	Ja, oké. En op die […] afbeelding staat bijvoorbeeld wel *unne dame*?
	‘Yes, alright. But that […] image [prompt], for example, does show *unne dame*?’
Y2C:	Ja, maar da is omdat ’t *unne dame* is.
	‘Yes, but that’s because *unne dame* is the correct form.’

In the fifth focus group, we see a revealing example of a participant not relying on his own dialect use but on his knowledge of another speaker’s dialect use (see also Section 4.2.2). Participant Y5D (an L2 dialect speaker) quotes his grandmother who speaks ‘very Brabantish’ and compares the dialect use in the prompts to her dialect use. As a result, he invents a norm on the spot, which entails alternating between marked and unmarked adnominals. Obviously, this invented norm does not make any sense considering the genuine grammar rule, but it does show some gut feelings of ‘enoughness’ (cf. Blommaert and Varis 2013), i.e., in order to be ratified as an authentic member of the Brabantish community, one should avoid the risk of overshooting the target. This can be done by using the suffix deliberately and not too frequently (see also Section 4.2.3 on identity work).

**Table:** 

Y5D:	Nou, mijn oma praat heel Brabants, en ik denk ik vergelijk zeg maar […] ook een beetje van zou oma da kunnen zeggen […] en ik denk dat zij minder snel twee keer in een zin *munne* zou zeggen en gewoon zou afwisselen tussen *munne* en *mun*.
	‘Well, my grandmother speaks very Brabantish, and I also compare a bit […] could grandma say that […] and I think she would be less inclined to say *munne* twice in a sentence and she would just alternate between *munne* and *mun*.’

#### 4.2.2 Knowledge reflected in the ratings

The focus group discussions reveal many inter-group and inter-individual differences for the younger participants, whereas the older participants are on the same page in their rejections of hyperdialectisms. For example, the younger participants in the first group mostly rely on their friends’ use of *unne*, being uncertain about their own use of the suffix. However, they do agree that it *is* Brabantish, and that other speakers’ use may be a trigger for their own use, which is, for example, reflected in the statements of participants Y1C and Y1E (both L2 dialect speakers).

**Table:** 

Y1E:	Ik denk dat ik *unne* best wel vaak hoor bij mijn vrienden ofzo.
	[…]
	Misschien gebruik ik het wel, ik heb echt niet in de gaten als ik het zou gebruiken. Maar ik heb wel in de gaten dat mijn vrienden het weleens zeggen. Maar het is *wel* Brabants.
	‘I think I hear *unne* quite often with my friends or something like that.’
	[…]
	‘I might use it, but I don’t really notice if I would use it. But I do notice when my friends use it. However, it *is* Brabantish.’
Y1C:	Ik weet niet of ik het echt zo zou gebruiken maar misschien inderdaad op een feestje ofzo als je met mensen bent die ook zo praten dan ga ik er vanzelf ook meer naartoe neigen of zoiets. Maar niet dat ik het echt zeg maar zelf zou gebruiken denk ik.
	‘I don’t know if I would really use it like that [the prompt], but maybe indeed at a party or something like that, if you are with people who also talk like that, I automatically start to lean more towards it or something like that. But it’s not like I would really say it myself, I think.’

In the second group of younger speakers, something interesting happens when one of the participants spontaneously starts reflecting on the audio recordings from the acceptability judgment task. Speaker Y2C (who reported a variety between the dialect and Dutch as his L1) considered the audio stimuli somewhat 'exaggerated'. However, the participants in this group, including Y2C, do not consider the prompt (i.e., Figure 3) exaggerated (although it contains a similar hyperdialectal form as the audio recordings), because they indicate to produce the form *unne dame* themselves, for example in their language use at home, which is revealed in their statements. Unlike the speakers in the first group, these speakers thus rely on their own dialect use.

**Table:** 

R:	Jij [Y2C] zei net van die opname dat je het overdreven vond. Hebben jullie dat bij dit ook, bij dit taalgebruik, of niet of minder?
	‘You [Y2C] just said that you thought the recording was exaggerated. With respect to this language use [the prompt], do you think the same, or not or not so much?’
Y2B:	Nou, ik heb ’t hier wa minder omdat ik ’t nu zelf kan zeggen …
	‘Well, I think it’s less [exaggerated] here because now I can use it myself …’
Y2C:	En ik vond meer tijdens de spraakberichten […] ze probeerden ’t té goed zeg maar te doen.
	[…] Da merk je gewoon hoe ’ie ’t zei. En hier zou je het inderdaad dus gewoon zelf kunnen zeggen.
	‘I thought they tried too hard during the audio recordings, you could just tell by how he pronounced it, and here you could just use it yourself, indeed.’
Y2E:	[…] Dit zou ik thuis ook wel kunnen zeggen.
	‘I could use this at home.’

Also in the third group of younger speakers, the prompt *sounds* Brabantish to the participants. Not only the use of the gender suffix in *unne dame*, but also another linguistic feature, i.e., the verb conjugation and the personal pronoun *bende gij* ‘are you’ particularly make the language recognizably Brabantish. In their judgments, participants Y3A, Y3C, and Y3D (all L2 dialect speakers) rely on their knowledge of other speakers’ dialect use instead of their own, as they report to consider *unne dame* as something they hear in in-group settings (i.e., with peers).

**Table:** 

Y3A:	Klinkt wel Brabants voor mij.
	‘It sounds Brabantish to me.’
Y3C:	Voor mij is die *bende gij* in de eerste zin heel Brabants. Die herken ik wel.
	‘For me, *bende gij* “are you” in the first sentence sounds very Brabantish. I do recognize that one.’
Y3D:	En ook *unne dame*.
	[…]
	Dit is iets wat je in een groep ofzo zou horen.
	‘And also *unne dame*.’
	[…]
	‘This is something you would hear in a group or so.’

In the fourth group of younger speakers, inter-individual differences are clearly visible in the in-group negotiation, with some speakers relying on their own use of the suffix as well as some speakers relying on other speakers’ use of the suffix. Please note that some of them are still discussing the hyperdialectal use of *munnen* ‘my-m.sg’, from a previous prompt. Speaker Y4A uses the suffix himself and hears other people around him using it; speaker Y4B does not use the suffix and also considers it to be ‘exaggerated’; speaker Y4C only uses the suffix in a non-serious tone of voice; speaker Y4D knows very few people who use the suffix; and speaker Y4E cannot use the suffix himself but does recognize it from other speakers. Note that within this group, participants Y4A, Y4B, Y4C and Y4D are all L2 dialect speakers, and that only participant Y4E reported a variety between the dialect and Dutch as his L1.

**Table:** 

Y4B:	*Munnen* zou ik nooit schrijven.
	‘I would never write down *munnen*.’
R:	Maar wel zeggen?
	‘But would you say it?’
Y4A:	Jawel.
	‘Yes.’
Y4C:	Maar nie serieus. Ik zou da echt nie serieus gebruiken.
	‘But not seriously. I really wouldn’t use that seriously.’
Y4E:	Maar je hoort ’t wel. […] Maar das hetzelfde met *unne*, dat zeg je ook niet, ik zeg ook *un huis* niet *unne huis*.
	‘But you do hear it around you. […] That’s also the case with *unne*, you don’t use that yourself, I use *un huis* ‘a house’ [n], not *unne huis*.’
Y4B:	Nee *munne* is gewoon overdreven.
	‘No, *munne* is just over the top.’
Y4D:	Ik ken maar weinig mensen die echt *munne* in een zin zouden zeggen.
	‘I know very few people who would actually use *munne* in a sentence.’
Y4A:	*Munne maot*, weet je wel, da hoor je echt wel af en toe.
	‘*Munne maot* “my buddy” [m], you really do hear that sometimes.’

The fifth group of younger speakers also shows inter-individual variation in their reported use of the suffix, e.g., comparing participant YA5 (an L2 dialect speaker who does not use the suffix) with participant Y5C (an L2 dialect speaker who recognizes it from others) and participant Y5D (an L2 dialect speaker who uses the suffix himself). Interestingly, participant Y5D comes up with a different truncated form of the indefinite article, spelled out as *nen*, to which the other speakers, even Y5A, agree. Note that this truncated form also occurs in traditional dialect use (as illustrated, for example, by the statements of the older participants below), but again only with singular masculine nouns.

**Table:** 

R:	Hier staat ook weer zo’n lidwoord in, *unne dame*, wat vinden jullie daarvan?
	‘Here again there is such an article, *unne dame*, what do you guys think?’
Y5A:	Ik zou het zelf niet gebruiken in ieder geval.
	‘I wouldn’t use it myself in any case.’
	[…]
Y5C:	Ik heb die wel vaker gehoord.
	‘I have heard that one before.’
Y5D:	Ik zou misschien eerder zeggen zelfs in plaats van *unne* misschien […] *nen dame* ofzo.
	‘Instead of *unne*, I would perhaps rather say *nen dame* or something like that.’
Y5A:	*Nen* vind ik dan weer wel kunnen […] das denk ik ook omdat ’t meer voorkomt en je vaker hoort ofzo.
	‘I think *nen* is acceptable […] I think that’s because it’s more common and you hear it more often or something.’

The quantitative results of the acceptability judgment task predict different statements from the older speakers, who are probably more inclined to rigorously reject any hyperdialectism. This is also shown in the focus group data. For example, participants in the first group of older speakers (all L1 dialect speakers) immediately respond to the incorrect use of the suffix, both relying on their own grammar knowledge as on the use of others. The hyperdialectal form ‘is not possible’, ‘makes no sense’, and ‘nobody uses it’. Instead, they come up with the traditional form without the suffix: *’n dame*.

**Table:** 

O1E:	*Unne dame?* ’k Zou nie weten, in heel Brabant, wie zegt *unne dame*?
	‘*Unne dame*? I wouldn’t know in whole Brabant, who says *unne dame*?’
O1D:	’n! ’n!
	‘[It’s] *’n*!’
O1C:	’n! Zoiets vraogde nie aan *’n dame*.
	‘[It’s] *’n*! *Zoiets vraogde nie aan ’n dame* “You do not ask something like that to a lady”.’
O1D:	Aan *’n dame*.
	‘To *’n dame*.’
O1B:	Mar *unne*, da geloof ik nie da iemand da zegt.
	‘But *unne*, I think nobody uses that.’
O1E:	Ken nie! Gó nergens over!
	‘It’s not possible! It makes no sense!’

This pattern occurs in every group of older speakers, with the hyperdialectal form being constantly corrected into the traditional dialect form, and participants also using dismissive judgments such as ‘wrong’ and ‘disruptive’, as is for example shown in the following excerpt of the second group (again all participants reported to be L1 dialect speakers).

**Table:** 

O2C:	Hé, dòr stò wir *’ne dame*.
	‘Hey, it shows again *’ne dame*.’
	[…]
	Dès in ieder geval fout.
	‘That’s wrong in any case.’
O2B:	Nee, ’t is aalt *’n*.
	‘No, it’s always *’n*.’
O2C:	*’ne dame*, dè vin ’k storend.
	‘*’ne dame*, I think that’s disruptive.’

#### 4.2.3 Identity work

The final topic pertains to the question whether hyperdialectisms are conscripted for identity service by the younger speakers who haven’t completely acquired it as an L2, but who want to portray themselves as members of the Brabantish community. Conversely, the question arises whether older speakers who reject the hyperdialectisms on grammatical grounds believe that these forms hinder an authentic Brabantish image. The statements from the older participants reveal that they consider hyperdialectisms as resulting from ‘make-shift’ speakers who do not master the traditional dialect but still want to *sound* Brabantish. However, these bogus speakers fail in their attempt, as the older participants regard the hyperdialectisms as inauthentic, which is for example shown in the following statements retrieved from the third focus group (all L1 dialect speakers).

**Table:** 

O3E:	Ja, die wullen dan dè eigenlijk ’n bietje Brabants laten lijken, hè?
	‘Yes, they want to make it look Brabantish, right?’
R:	Maar komt dit voor jullie allemaal niet authentiek over […]?
	‘But does this not seem authentic to you?’
O3D:	Nee!
	‘No!’
O3E:	Nee, komt voor mij niet – abseluut niet authentiek over. Nee, zeker nie.
	‘No, it definitely doesn’t seem authentic to me. No, definitely not.’
O3B:	Dit? Authentiek, nee. Nee.
	‘This? Authentic, no. No.’

The older speakers of the fifth focus group (two L2 and three L1 dialect speakers) even consider the use of hyperdialectisms as offensive. Participant O5E ascribes it to people from outside the province, whom are called *Bovensloters* (lit. ‘people from above the ditch’), i.e., people from above the rivers *Rijn/Waal* and *Maas* who speak distinct dialects. In addition, speaker O5B, who was previously mentioned as a very proficient L2 dialect speaker, attributes the use of hyperdialectisms to younger speakers who speak an ‘artilect’, an artificial dialect.

**Table:** 

O5B:	D-d-d-dit is echt ’n belediging van Brabant.
	‘This is really an insult to Brabant.’
O5C:	Iemand van boven de rivieren die toch mee wil doen.
	‘It’s someone from above the rivers who still wants to be included.’
O5E:	’n Bovenslòtse.
	lit. ‘someone from above the ditch’
O5B:	OF! ’t Kan ok zijn … ded ’t ’n jong iemand is … die ’n sort artilect het, zal ik mar zeggen, dus ’n kunstmaotig dialect.
	‘OR! It can also be the case that it’s a young person who speaks an “artilect”, so to say, an artificial dialect.’

Also for the older speakers in the second focus group, hyperdialectisms are reminiscent of younger speakers who did not acquire the traditional dialect grammar, as they are often raised in Dutch (which is also the case for the younger participants in our study). However, they think it is a matter of these speakers ‘not knowing any better’ (cf. Trudgill 1988).

**Table:** 

R:	En denken jullie dat dit voor jongeren kan werken? Dus dat die dit wel […] echt Brabants vinden?
	‘And do you think this can work for young people? That they consider it real Brabantish?’
O2F:	Nou, ik denk dè ze’r weinig benul van hebben.
	‘Well, I think they have little clue.’
O2C:	As ge’r nie himmel mee opgegroeid bent, dan denk ik dè ge ok nie mee ziet dè *’ne dame* nie klopt.
	‘If you didn’t grow up with it at all, then I guess you also don’t notice that *’ne dame* is not correct.’

In the first focus group, older speakers argue that producing hyperdialectisms may also be the result of speakers trying to stand out, for example to convey the convivial and cozy character that is associated with the Brabantish culture (cf. Doreleijers 2023; Doreleijers and Swanenberg 2023a). For the older speakers, this could actually be counterproductive, because to them real Brabantishness can only be achieved through traditional dialect use.

**Table:** 

R:	Worrom zo iemand dè doen […]?
	‘Why would anyone do that?’
O1D:	Om op te vallen.
	‘To stand out.’
O1E:	Die kent gin Brabants!
	‘That person doesn’t know Brabantish!’
O1D:	Ja, maar dit is ook geen Nederlands!
	‘Yes, but it’s not Dutch either!’
O1C:	’t Is fout.
	‘It’s wrong.’
O1B:	Nee, maar hij doet ’t, denk ik, om eh, de Brabantse gezelligheid uit te willen stralen.
	‘No, but I think he also uses it to convey Brabantish coziness.’
O1C:	Dès wel ’n punt, ja.
	‘That’s a good point, yes.’
O1E:	Ja. Mer dan kan ok averechts effect hebben!
	‘Yes. But it can also be counterproductive.’

However, for the younger speakers, the use of hyperdialectisms is often part of stylistic practices in which they want to portray themselves as ‘Brabantish’, for example when people ask them if they come from Brabant. Interestingly, in the excerpt below, speaker Y4A (an L2 dialect speaker) adds that this dialect use is often embedded in an exaggerated context, with a non-serious tone of voice.

**Table:** 

R:	En hebben jullie zelf een gevoel bij wanneer je dat dan wel zou gebruiken, wanneer zou je *unne* zeggen in plaats van *un* en wanneer zou je *den* zeggen in plaats van *de*?
	‘And do you have a feeling about when you would use it, when you would use *unne* instead of *un* and when you would use *den* instead of *de*?’
Y4D:	Als mensen vragen “kom je uit Brabant?” en ik wil dat duidelijk maken.
	‘When people ask “are you from Brabant?” and I want to make that clear.’
Y4A:	Ja ook weer in zo’n overdreven Brabantse zin die dan vaak onserieus is.
	‘Yes, another of those exaggerated Brabantish phrases that are often not intended to be taken seriously.’

Interestingly, younger speakers from the first focus group think the suffix could also come across as ‘forced’ (trying too hard) when it is used by peers (they use one of the prompts with a neuter hyperdialectism *munne dialect* ‘my-m.sg dialect.n.sg’ as an example here). They mainly ascribe the use of this feature to older, traditional dialect speakers, in this case the stereotypical old Brabantish farmer, but also to people who pretend to be Brabantish, although one would expect both speakers to produce quite opposite forms in terms of traditional grammar. In other words, the younger speakers are not able to distinguish between grammatical and ungrammatical forms, which is supported by the quantitative results, but rather base their acceptability judgments of the suffix on its perceived (speaker or situational) appropriateness. For example, the same feature may be perceived as funny in one context, but as exaggerated (or even annoying) in another, especially when the feature is appropriated by a non-Brabantish speaker whose usage actually shows they do *not* belong to the community.

**Table:** 

Y1A:	Ik vind mezelf redelijk Brabants maar als ik tegen iemand ga zeggen ‘was munne buuk maar net zo plat als munne dialect’ dan denken die ook van wat ben je nou aan het forceren.
	‘I consider myself fairly Brabantish, but if someone says to me *was munne buuk maar net zo plat als munne dialect* then I also think why are you “forcing” it like that.’
R:	Je vindt da wel geforceerd dus overkomen?
	‘You think it comes across as “forced”?’
Y1A:	Ik denk, ja oké ligt eraan als een ouwe boer, dit tegen mij zegt, een ouwe Brabantse boer, dan kan ik het nog accepteren maar over het algemeen als vrienden di tegen mij zeggen dan denk ik echt wa ben je aan ’t doen.
	‘It depends, I think, if an old farmer, an old Brabantish farmer, says this to me, then I would accept it, but in general when friends say this to me, I am really wondering like what are you doing.’
	[…]
Y1A:	Maar dan zegt iemand het ook gewoon om Brabants te willen zijn.
	‘But then someone says it just because they want to be Brabantish.’
Y1D:	Dat is iemand die ergens anders woont …
	‘This is someone who lives somewhere else …’
Y1A:	Ja, precies.
	‘Yes, exactly.’
Y1D:	Naar Eindhoven komt en dan dat zegt om te forceren maar dan denkt iedereen nou jij komt dus precies niet hier vandaan.
	‘Someone who comes to Eindhoven and says this “to force” [their identity] but then everybody thinks well you’re definitely *not* from here.’

However, in specific (informal) contexts, ‘exaggerated talk’ may be the desired talk. For example, the younger speakers in the fifth focus group reflect on their dialect use with friends while trying ‘to be funny’. This turns out to be an ultimate and legitimate context to produce hyperdialectisms, without the speakers being ‘out of order’ in the presence of their peers (cf. Swanenberg 2019).

**Table:** 

Y5B:	Zo zou ik ‘t eigenlijk nooit horen behalve als je misschien met vrienden ofzo een beetje aan ’t praten bent […] soort van een beetje heel erg overdreven ofzo aan ’t praten.
	‘I would never actually hear it like that except maybe if you’re talking to friends or something, kind of talking very exaggerated or something like that.’
R:	Ja. En wanneer in wat voor situatie zou je overdreven gaan praten dan? Kan je een voorbeeld van geven?
	‘Yes. And when or in what kind of situation would you start talking in an exaggerated way then? Can you give an example of that?’
Y5B:	Ja als we gewoon een beetje grappig aan het doen zijn ofzo.
	‘Yes, when we are just acting a bit funny or something.’
	[…]
Y5D:	Ik merk hetzelfde ook wel denk ik met vrienden ooit, heel overdreven en dat is dan grappig.
	‘I also notice the same thing, I think, with friends sometimes, very exaggerated and that’s funny.’

In general, the younger speakers seem to have different associations, i.e., indexical values (cf. Eckert 2008) when it comes to talking Brabantish, i.e., they are not fixed but rather variable, ranging from their grandparents’ language to talking with friends in the pub, at a festival or at a campsite, as is for example shown by the following statement of L2-dialect speaker Y5D.

**Table:** 

Y5D:	Ik weet nie echt hoe ik da moet uitleggen, ja opa en oma spreken ook zo […] maar dat komt ook doordat zij oud zijn denk ik dat ze zo praten en misschien zijn die cafés en festivals gezellig en de camping is ook zo’n sfeertje zeg maar en dan ga je automatisch meer Brabants praten door de vibe zeg maar, dat het gezellig is.
	‘I don’t really know how to explain it, yes grandpa and grandma also talk like that, but that’s also because they are old, I think, that’s why they talk like that, and maybe those pubs and festivals are cozy and at the campsite there’s the same ambience, and then you automatically start talking more Brabantish because of the vibe so to say, it’s cozy.’

Building on the last statement, it seems that speaking Brabantish for the younger participants does not consist of adhering to a strict system of rules. Instead, talking Brabantish is ‘a vibe’, regardless of its correctness. The use of the gender suffix, whether it is used in a traditional or a hyperdialectal way, contributes to Brabantishness and can be deployed in specific circumstances when younger speakers want to claim their local identity, albeit often in a funny way, e.g., as ‘slang’ indexing a non-adult subcultural identity (cf. Tsiplakou 2011).

### 4.3 Discussion of the qualitative results

The qualitative materials provide answers to RQ3 and its sub questions. The quantitative data revealed that younger L2 dialect speakers are more tolerant for the ungrammatical variants than older L1 dialect speakers. The qualitative data confirm that this is due to a lack of proficiency and meta-grammatical awareness, with younger participants both relying on their own usage as well as on what they *think* they know about other speakers’ usage. Moreover, younger speakers are more inclined to accept incorrect forms because they *sound* Brabantish, though this only seems to be the case for in-group contexts. On the contrary, out-group members producing hyperdialectisms are rather evaluated as fake (i.e., make-shift, artificial, or forced), by both the younger and the older participants.

The frequent use of hyperdialectal gender suffixes on social media (Doreleijers 2023), and the quantitatively and qualitatively confirmed tolerance for and embracing of these forms in the current study, suggest that they are much more than passive first-order indexes. All the evidence collected confirms that hyperdialectisms are consciously manipulated second-order, if not third-order indexes. This is an interesting finding in a number of ways. To begin with, it is widely accepted in the (Labovian) sociolinguistic community that syntactic forms are not typically used for social (identity) work, because they are entrenched too deeply in the linguistic motor of language to be consciously exploitable (but see, for example, Grondelaers et al. [2022] and Moore [2021] for counterevidence). However, the current study shows that the gender suffix is in fact used for the purpose of social meaning profiling. Importantly, speakers ‘violating’ the traditional dialect grammar seem to have come up with new norms (cf. Doreleijers et al. 2023). This nicely illustrates that grammatical features can be reinterpreted, and subsequently used to express social meaning.

## 5 Conclusions

The current paper investigated the perception of hyperdialectisms by younger and older speakers from the region of Eindhoven, a city in the southern Dutch province of North Brabant. In this area, processes of dialect leveling and loss have resulted in structural change in the use of salient dialect features. Previous research (Doreleijers et al. 2020), more concretely, has revealed that the use of the adnominal gender suffix is undergoing significant change in younger L2 dialect speakers: the gender suffix is no longer only tied to masculine singular gender, but to all genders, and sometimes even plurals, leading to hyperdialectisms. The aim of this study was to examine whether the variation found in production data is also reflected in perceptual hyperdialectism (Jamieson 2020), and whether there is a difference between younger and older evaluations.

From acceptability judgment ratings by 46 Brabantish respondents, and these respondents’ subsequent qualitative statements obtained in focus group discussions, we have learned that hyperdialectisms are rated as less acceptable than the correct (traditional) dialect forms, and that this effect is mostly restricted to the older participants, who are self-reported L1 speakers, in contrast to the younger speakers who generally reported to have acquired the dialect as an L2.

The qualitative statements complement and stratify the scaled ratings by showing that older respondents categorically disapprove of hyperdialectisms. They tend to consider these forms as wrong, artificial, and annoying. Interestingly, the younger respondents’ acceptance of hyperdialectisms is not merely the result of incomplete dialect acquisition and limited knowledge of the traditional grammar, but also, and crucially, of conscious local identity-building. According to the qualitative statements, the gender feature is not only noticed by the younger speakers, but also regarded as emblematic for the Brabantish community. The suffix, in other words, is loaded with second and third-order indexical meaning, since speakers are able to use it in practices of stylization, i.e., when they want to portray their ‘Brabantishness’. However, this is only ‘allowed’ for in-group members, as outsiders sporting hyperdialectisms are dismissed as fake or forced.

It goes without saying that this study suffers from a number of shortcomings. In view of the fact that we carried out extensive quantitative and qualitative analysis with the same respondents, our participant sample is inevitably small. In addition, our group recruitment of respondents – via schools and dialect organizations – engendered some imbalances: most crucially, the L1-variable is not well-proportioned across the age groups: while most of our older respondents were native dialect speakers, almost no younger respondents were. This has complicated the statistical analysis somewhat. Also, the plural forms are the only category that includes the negative form *ginne* ‘no’. Since the indefinite article *ene(n)* ‘a’, central to the study (among the singular forms), cannot be combined with plurals, this was the most comparable option, but not optimal. Future research could therefore compare other conditions, for example including the definite article (that is used with both singular and plural nouns). Future designs could also incorporate comparisons in judgments between ‘incorrect’ and ‘correct’ forms (i.e., with and without the suffix) in feminine, neuter, and plural contexts.

On a methodological note, we nonetheless hope to have convinced the reader that our confrontation of quantitative and qualitative methods is the optimum tool to extract grammatical preference, meta-grammatical knowledge, and regional attitude data. While it would in principle have been possible to elicit the latter two types of data in a more controlled experimental follow-up to the acceptability judgment task, there are a number of disadvantages to be considered. Apart from the obvious fatigue and boredom side effects of a second rating task, it would be a challenge to separate evaluation of (the appropriateness of) the grammatical variable from holistic assessments of the general ‘Brabantishness’ of the stimuli. The main advantage of this second evaluation task, viz. obtaining deeper and more reliable attitudes by enforcing respondent ignorance (Rosseel and Grondelaers 2019), would in any case be thwarted by the fact that respondents are fully aware of the variable of interest by the time the second experiment starts. In view of this unavoidable awareness, we believe that small discussion groups of friendly peers offer the best guarantee for obtaining spontaneous but focused, uninhibited attitudes. Our own confrontation of quantitative and qualitative methods has at any rate provided clear evidence for a shift of the gender suffix towards social meaning at the expense of grammatical function.

## Abbreviations

m: Masculine
f: Feminine
n: Neuter
sg: Singular
pl: Plural

## Appendix

**Table:**

*Number*	*Score*					*Total*
	1	2	3	4	5	
1_Plu	4	11	11	13	7	46
	8.7 %	23.9 %	23.9 %	28.3 %	15.2 %	100 %
2_SingN	1	10	14	19	2	46
	2.2 %	21.7 %	30.4 %	41.3 %	4.3 %	100 %
3_SingF	1	6	6	15	18	46
	2.2 %	13 %	13 %	32.6 %	39.1 %	100 %
4_SingF	0	7	3	16	20	46
	0 %	15.2 %	6.5 %	34.8 %	43.5 %	100 %
5_SingN	4	5	6	14	17	46
	8.7 %	10.9 %	13 %	30.4 %	37 %	100 %
6_Cor	0	0	0	14	32	46
	0 %	0 %	0 %	30.4 %	69.6 %	100 %
8_Cor	2	4	6	20	14	46
	4.3 %	8.7 %	13 %	43.5 %	30.4 %	100 %
9_SingF	5	6	6	16	13	46
	10.9 %	13 %	13 %	34.8 %	28.3 %	100 %
10_Plu	3	15	8	13	7	46
	6.5 %	32.6 %	17.4 %	28.3 %	15.2 %	100 %
12_SingF	3	9	9	13	12	46
	6.5 %	19.6 %	19.6 %	28.3 %	26.1 %	100 %
14_SingN	6	10	13	10	7	46
	13 %	21.7 %	28.3 %	21.7 %	15.2 %	100 %
15_Cor	0	1	8	17	20	46
	0 %	2.2 %	17.4 %	37 %	43.5 %	100 %
17_SingN	8	9	13	9	7	46
	17.4 %	19.6 %	28.3 %	19.6 %	15.2 %	100 %
19_SingF	6	8	8	15	9	46
	13 %	17.4 %	17.4 %	32.6 %	19.6 %	100 %
20_Cor	0	1	3	14	28	46
	0 %	2.2 %	6.5 %	30.4 %	60.9 %	100 %
21_SingN	3	4	13	10	16	46
	6.5 %	8.7 %	28.3 %	21.7 %	34.8 %	100 %
24_SingF	7	6	5	15	13	46
	15.2 %	13 %	10.9 %	32.6 %	28.3 %	100 %
25_Plu	1	5	8	17	15	46
	2.2 %	10.9 %	17.4 %	37 %	32.6 %	100 %
26_SingN	10	10	9	11	6	46
	21.7 %	21.7 %	19.6 %	23.9 %	13 %	100 %
27_Cor	0	5	6	15	20	46
	0 %	10.9 %	13 %	32.6 %	43.5 %	100 %
28_SingN	4	8	5	17	12	46
	8.7 %	17.4 %	10.9 %	37 %	26.1 %	100 %
29_Cor	2	7	13	10	14	46
	4.3 %	15.2 %	28.3 %	21.7 %	30.4 %	100 %
30_SingN	5	8	7	11	15	46
	10.9 %	17.4 %	15.2 %	23.9 %	32.6 %	100 %
31_SingN	6	9	7	15	9	46
	13 %	19.6 %	15.2 %	32.6 %	19.6 %	100 %
32_Plu	15	14	9	7	1	46
	32.6 %	30.4 %	19.6 %	15.2 %	2.2 %	100 %
33_SingN	6	9	9	17	5	46
	13 %	19.6 %	19.6 %	37 %	10.9 %	100 %
34_SingN	4	7	10	15	10	46
	8.7 %	15.2 %	21.7 %	32.6 %	21.7 %	100 %
35_Plu	1	7	8	18	12	46
	2.2 %	15.2 %	17.4 %	39.1 %	26.1 %	100 %
36_SingN	7	15	7	12	5	46
	15.2 %	32.6 %	15.2 %	26.1 %	10.9 %	100 %
37_Plu	8	15	6	14	3	46
	17.4 %	32.6 %	13 %	30.4 %	6.5 %	100 %
38_Cor	2	10	4	11	19	46
	4.3 %	21.7 %	8.7 %	23.9 %	41.3 %	100 %
39_SingF	5	10	6	8	17	46
	10.9 %	21.7 %	13 %	17.4 %	37 %	100 %
40_Cor	0	0	2	10	34	46
	0 %	0 %	4.3 %	21.7 %	73.9 %	100 %
41_SingN	8	7	3	16	12	46
	17.4 %	15.2 %	6.5 %	34.8 %	26.1 %	100 %
42_SingF	8	10	10	10	8	46
	17.4 %	21.7 %	21.7 %	21.7 %	17.4 %	100 %
43_Plu	11	11	13	9	2	46
	23.9 %	23.9 %	28.3 %	19.6 %	4.3 %	100 %
44_SingF	5	5	11	15	10	46
	10.9 %	10.9 %	23.9 %	32.6 %	21.7 %	100 %
46_Cor	1	2	1	24	18	46
	2.2 %	4.3 %	2.2 %	52.2 %	39.1 %	100 %
47_Plu	4	16	10	8	8	46
	8.7 %	34.8 %	21.7 %	17.4 %	17.4 %	100 %
48_Plu	4	7	9	13	13	46
	8.7 %	15.2 %	19.6 %	28.3 %	28.3 %	100 %
50_Cor	3	5	6	17	15	46
	6.5 %	10.9 %	13 %	37 %	32.6 %	100 %
51_Cor	0	2	7	15	22	46
	0 %	4.3 %	15.2 %	32.6 %	47.8 %	100 %
52_Plu	8	15	12	7	4	46
	17.4 %	32.6 %	26.1 %	15.2 %	8.7 %	100 %
53_SingF	2	3	6	20	15	46
	4.3 %	6.5 %	13 %	43.5 %	32.6 %	100 %
54_Plu	11	11	9	8	7	46
	23.9 %	23.9 %	19.6 %	17.4 %	15.2 %	100 %
55_SingF	8	6	10	14	8	46
	17.4 %	13 %	21.7 %	30.4 %	17.4 %	100 %
56_Plu	8	16	9	10	3	46
	17.4 %	34.8 %	19.6 %	21.7 %	6.5 %	100 %
57_SingF	5	6	9	15	11	46
	10.9 %	13 %	19.6 %	32.6 %	23.9 %	100 %
58_Plu	9	10	14	6	7	46
	19.6 %	21.7 %	30.4 %	13 %	15.2 %	100 %
59_Cor	1	2	7	17	19	46
	2.2 %	4.3 %	15.2 %	37 %	41.3 %	100 %
61_Cor	1	6	8	9	22	46
	2.2 %	13 %	17.4 %	19.6 %	47.8 %	100 %
62_SingN	7	14	8	11	6	46
	15.2 %	30.4 %	17.4 %	23.9 %	13 %	100 %
63_SingN	7	8	10	13	8	46
	15.2 %	17.4 %	21.7 %	28.3 %	17.4 %	100 %
64_SingF	6	10	4	13	13	46
	13 %	21.7 %	8.7 %	28.3 %	28.3 %	100 %
65_Cor	0	11	11	14	10	46
	0 %	23.9 %	23.9 %	30.4 %	21.7 %	100 %
66_SingF	6	7	2	19	12	46
	13 %	15.2 %	4.3 %	41.3 %	26.1 %	100 %
67_Plu	5	8	12	12	9	46
	10.9 %	17.4 %	26.1 %	26.1 %	19.6 %	100 %
68_SingN	8	10	8	13	7	46
	17.4 %	21.7 %	17.4 %	28.3 %	15.2 %	100 %
69_Plu	6	15	12	11	2	46
	13 %	32.6 %	26.1 %	23.9 %	4.3 %	100 %
70_Cor	1	5	5	17	18	46
	2.2 %	10.9 %	10.9 %	37 %	39.1 %	100 %
71_SingF	6	7	2	12	19	46
	13 %	15.2 %	4.3 %	26.1 %	41.3 %	100 %
73_Cor	0	1	3	12	30	46
	0 %	2.2 %	6.5 %	26.1 %	65.2 %	100 %
75_Plu	9	9	11	7	10	46
	19.6 %	19.6 %	23.9 %	15.2 %	21.7 %	100 %
76_SingF	7	9	10	8	12	46
	15.2 %	19.6 %	21.7 %	17.4 %	26.1 %	100 %
***Total***	294	505	500	846	799	2,944
	10 %	17.2 %	17 %	28.7 %	27.1 %	100 %

*χ*^2^ = 716.769, df = 252, Cramer’s *V* = 0.247, *p* = 0.000.
